# Retraction Note: Clinical and immuno-proteomic approach on *lantana camara* pollen allergy—a major health hazard

**DOI:** 10.1186/s13223-024-00946-z

**Published:** 2025-01-02

**Authors:** Kavita Ghosal, Bodhisattwa Saha, Swati Gupta Bhattacharya

**Affiliations:** Division of Plant Biology, Bose Institute, Main Campus, 93/1, A.P.C. Road, 700009 Kolkata, West Bengal India

**Retraction Note to: Allergy Asthma Clin Immunol (2016) 12:33**

10.1186/s13223-016-0135-z

The Editor-in-Chief is retracting this article after concerns were raised about the data reported. Specifically, there appear to be areas of duplication and other irregularities in several of the immunoblots presented in Fig. 2a. The authors were unable to provide raw data for this figure upon request by the Publisher. The Editor-in-Chief no longer has confidence in the reliability of this article’s findings and conclusions.

Kavita Ghosal and Swati Gupta Bhattacharya did not explicitly state whether they agree or disagree this retraction. Bodhisattwa Saha has not responded to correspondence from the editor or publisher about this retraction.

